# Childhood and Adolescent Factors and Thyroid Cancer Incidence in Adult Women in the Sister Study Cohort

**DOI:** 10.1007/s10552-025-02085-1

**Published:** 2026-02-20

**Authors:** Thi-Van-Trinh Tran, Katie M. O’Brien, Rebecca Troisi, Dale P. Sandler, Cari M. Kitahara

**Affiliations:** 1 Radiation Epidemiology Branch, Division of Cancer Epidemiology and Genetics, National Cancer Institute, National Institutes of Health, 9609 Medical Center Drive, Room 7E-456, Bethesda, MD 20892-9778 USA; 2 Epidemiology Branch, National Institute of Environmental Health Sciences, Research Triangle Park, Durham, NC USA; 3 Division of Cancer Epidemiology and Genetics, Trans-Divisional Research Program, National Cancer Institute, National Institutes of Health, 9609 Medical Center Drive, Room 7E-456, Bethesda, MD 20892-9778 USA

**Keywords:** Thyroid neoplasms, Incidence, Childhood exposures, Adolescent exposures, Risk factors, Observational study

## Abstract

**Purpose:**

Differentiated thyroid cancer (DTC) is more common in women than in men but the etiology is not well understood. We therefore investigated the association between childhood and adolescent factors and subsequent DTC incidence in women.

**Methods:**

We used data from 47,913 women enrolled (2003–2009) in the U.S. nationwide Sister Study cohort who were cancer-free at baseline. We used Cox regression models to assess associations of DTC incidence with self-reported baseline characteristics, including perceived body size, hormonal, lifestyle, and socioeconomic factors through age 20, adjusting for attained age (timescale), and race/ethnicity.

**Results:**

Over follow-up (median: 13.1 years), 239 DTC cases were identified. Factors associated with higher DTC incidence included being taller than peers at age 10 (hazard ratio [HR] = 1.41, 95% confidence interval [CI] = 1.06–1.89), being lighter (HR = 1.37, 95%CI = 0.97–1.91) or heavier (HR = 1.28, 95%CI = 0.96–1.71) than peers during teen years, and ever not having enough to eat during childhood (HR = 1.67, 95%CI = 1.15–2.43). DTC incidence was lower among those with childhood higher household educational level (HR_Bachelor’s degree or higher vs high school, GED or less_ = 0.75, 95%CI = 0.55–1.03). We did not find notable associations for other factors.

**Conclusion:**

Our findings suggest that childhood growth, nutrition, and socioeconomic factors may influence DTC incidence in women.

**Supplementary Information:**

The online version contains supplementary material available at 10.1007/s10552-025-02085-1.

## Introduction

Thyroid cancer ranks as the fifth most common cancer in women globally [1]. Few modifiable risk factors have been identified, apart from obesity and childhood exposure to ionizing radiation [2]. The slow growth of most thyroid nodules [3, 4], the relatively young age-at-diagnosis compared to other cancers [2], and the higher incidence in women compared to men starting from early adolescence [5] suggest that biological factors contributing to thyroid cancer development may originate at a very young age.

During childhood and adolescence, particularly during puberty, women experience significant changes in levels of sex steroid hormones [6], growth hormone [7], and insulin-like growth factor-1 (IGF-1) [8, 9], which contribute to sexual development, and changes in growth and body composition. These hormones have been shown to promote the growth of both benign and malignant thyroid cells [10–12], with thyroid tumors also commonly overexpressing estrogen [13], and insulin-like growth factor receptors [14]. Body size and reproductive factors during these developmental periods may either represent or serve as proxies for these early-life hormonal exposures and have been associated with several female-predominant cancers [15–17].

However, the scarcity of longitudinal studies with data on both early-life factors and thyroid cancer incidence makes it challenging to investigate these associations. To date, the few longitudinal studies examining early-life body size and thyroid cancer incidence have suggested positive associations for childhood and adolescent height and body mass index (BMI) [18–21], but most of them did not account for adult obesity at diagnosis [18–20]. Evidence on early-life reproductive factors is mostly limited to age at menarche, with inconsistent results [22–25], while data on other factors, such as age at breast development and start age for hormonal birth control, are scarce. Additionally, lifestyle (e.g., smoking) and socioeconomic factors known to influence hormone levels either directly or by increasing exposure to endocrine disruptors [26, 27], could also contribute to thyroid cancer development.

We aimed to investigate the associations of anthropometric, reproductive, lifestyle, and socioeconomic factors experienced during childhood and adolescence and thyroid cancer incidence using data from the large U.S. nationwide Sister Study cohort. We hypothesized that factors that reflect early-life exposure to higher levels of sex steroid hormones, growth hormone, and IGF-1 would be associated with higher rates of differentiated thyroid carcinoma (DTC), which accounts for 95% of all thyroid cancer cases. [28]

## Methods

### Study Population

The Sister Study is a U.S. nationwide prospective cohort of 50,884 women aged 35–74 at the time of enrollment (2003–2009) [29]. All participants had a sister with breast cancer but were breast cancer-free themselves at baseline. Baseline data were collected using self-administered questionnaires and computer-assisted telephone interviews. Anthropometric measurements and biospecimens were obtained at in-person home visits. Participants are recontacted every 2–3 years for health and lifestyle updates, with response rates consistently exceeding 85% [30]. All participants provided written informed consent and the National Institutes of Health’s Institutional Review Board approved the study. Data used here are through mid-September 2021 (data release 11.1).

We excluded individuals who had a history of invasive cancer (*n* = 2911) or reported receiving chemotherapy (for cancer, *n* = 30) or radiotherapy (for cancer, *n* = 25) before baseline, and those who withdrew from the study (n = 5). After exclusions, the study population comprised 47,913 individuals.

## Outcome Definition

By the end of follow-up (median 13.1 years, interquartile range, IQR 11.5–15), 252 first primary thyroid cancer diagnoses were reported. Medical records/pathology reports (*n* = 187) or National Death Index/death certificate report (*n* = 1) were available for 188 (74.6%) cases. To restrict the case group to DTCs, based on medical records and pathology reports, we excluded poorly differentiated thyroid carcinoma (confirmed with pathology reports; *n* = 5), anaplastic thyroid carcinoma (histology code: 8021; *n* = 1), medullary thyroid carcinoma (histology codes: 8346, 8347, 8510, *n* = 5), and thyroid cancer of indeterminate histology (histology code: 8265, 9084, *n* = 2). After exclusions, our analyses included incident 239 DTC cases, unless otherwise specified.

We further classified DTC cases with confirmed histology codes (*n* = 174) as papillary thyroid carcinomas (histology codes: 8050, 8260, 8340–8344, 8350, 8450–8460, *n* = 164), follicular thyroid carcinomas (histology codes: 8290, 8330–8335, *n* = 7), or unspecified carcinomas and neoplasms (histology code: 8000, 8010, *n* = 3).

## Exposure Definitions

Childhood and adolescent factors were ascertained from responses to baseline questionnaires. Anthropometry and reproductive factors included weight (lighter/same weight/heavier) and height (shorter/same height/taller) relative to peers at age 10, weight (lighter/same weight/heavier) relative to peers during teen years, age at breast development (continuous, categorized as < 11/11–13/ ≥ 14 years/unknown), age at menarche (continuous, categorized as < 12/12–13/ ≥ 14 years/unknown), age started using hormonal birth control (categorized as started before/after age 20/unknown). Responses indicating that age at breast development or menarche occurred after age 21 were reassigned as missing values.

Lifestyle factors included physical activity between ages 5 and 20 measured in average metabolic equivalent of task (MET)-hours per week (continuous, categorized as < 21/21- < 42/ ≥ 42 MET-hours/unknown; calculated based on the reported average weekly hours of sports/exercise activities done at least once a week for two or more months) [31], age started drinking regularly (started before/after age 20/unknown), average number of drinks per year in the years drinking regularly before age 20 (0/ < 60/60–229/ ≥ 230 drinks/unknown; calculated based on reported average drinks per week), [32] age started smoking (started before/after age 20/unknown), number of pack-years before age 20 (≤ 5/ > 5 pack-years), and total years of secondhand smoking under age 18 from caregiver or other household member (no secondhand smoking/ ≤ 10/ > 10 years/unknown).

Socioeconomic measures during childhood and adolescence included family income while growing up (well off/middle income/low income/poor/unknown), ever not having enough to eat during childhood (yes/no/unknown), highest household education level at age 13 (high school or GED or less/some college or associate or technical degree/bachelor’s degree or higher/unknown), family type at age 13 (two parents/single parent/unknown), and childhood urbanicity (urban or suburban/small town or rural areas/unknown).

Missing data were less than 5% for all variables except for number of drinks per year between ages 5 and 20 (9.3%). For categorical variables, we assigned missing data to the “unknown” category. For continuous variables, we imputed missing data using mean values.

## Baseline Covariates

Baseline examiner-measured BMI was calculated using in-person measurements of weight in kilograms divided by the square of height in meters. Demographic and socioeconomic characteristics, including age, self-identified race/ethnicity (non-Hispanic White/non-Hispanic Black/Hispanic/non-Hispanic all other races/unknown), personal attained education level (high school or GED or less/some college or associate or technical degree/bachelor’s degree or higher/unknown), and household annual income (< $50,000/$50,000-$99,999/ ≥ $100,000) were collected through computer-assisted telephone interviews.

## Statistical Analysis

We used Cox proportional hazards models to estimate hazard ratios (HRs) and 95% confidence intervals (CIs), with attained age as the time scale and self-reported race/ethnicity as a covariate. Follow-up time was calculated from age at baseline until age at DTC diagnosis, with censoring at age at first diagnosis of any other invasive cancer (excluding non-melanoma cancer), death, loss of follow-up, or mid-September 2021, whichever occurred first, unless otherwise specified. We assessed proportional hazards assumptions with plots of scaled Schoenfeld residuals against attained age, and formal testing included introducing an interaction term between exposures and attained age. No evidence of violation was found. We assessed non-linearity in the association between continuous variables and DTC incidence by visually inspecting plots of Martingale residuals against each variable and found no evidence of departure from linearity.

We considered health- and medical surveillance-related factors during adulthood as potential modifying factors for the associations between childhood and adolescent factors and DTC incidence. Therefore, we conducted stratified analyses by baseline BMI, personal attained education level, and household annual income. Tests for multiplicative interactions were conducted by including a cross‐product term in the model and evaluating the F‐test‐based P‐value. P‐values were two‐sided with an alpha of 0.05.

Risk factors for early onset (defined as diagnosis before age 50) and later-onset DTC may differ. Hence, we performed a secondary analysis assessing early- and late–onset DTCs separately. For the early-onset DTC analysis, we included only individuals under 50 at baseline, and censored them at 50 years. For the late-onset DTC analysis, follow-up time began at age 50 for individuals who turned 50 during the study period or age at baseline for those entering the study over age 50. We also performed analyses restricted to medically confirmed DTC cases and papillary thyroid carcinomas, separately. Additionally, we conducted complete-case analyses, excluding individuals with missing data on a model-specific basis. Lastly, we calculated E-values for both the observed association estimates and the limit of the confidence interval closest to the null. The E-value is defined as the minimum strength of association that an unmeasured confounder would need to have with both the exposure and the outcome, conditional on the measured covariates, to fully explain the observed associations. [33, 34]

Data analyses were conducted using SAS 9.4 and R version 4.3.1.

## Results

### Baseline Characteristics

Table 1 presents the baseline descriptive statistics. The median age at baseline was 55.4 years (interquartile range [IQR]: 48.9–62.1). Most women were non-Hispanic White (*n* = 39,947, 83.4%), and 61.7% (*n* = 29,566) had a BMI of 25 or greater. Over half of the participants held a bachelor’s degree or higher (*n* = 24,450, 51.0%), while 33.7% reported a household annual income of $100,000 or more (*n* = 16,161).

**Table 1:** Baseline characteristics of the Sister Study cohort (*n* = 47,913) and those diagnosed with differentiated thyroid cancer (DTC) during follow-up (*n* = 239)

Characteristic	Total cohort, *n* (%)	DTC cases, *N* (%)
Age (median, interquartile range)	55.4 (48.9, 62.1)	54 (47.4, 61.1)
*Self-identified race/ethnicity*		
Non-Hispanic White	39,947 (83)	207 (87)
Non-Hispanic Black	4304 (9)	9 (4)
Hispanic	2416 (5)	18 (8)
Non-Hispanic all other races	1231 (3)	5 (2)
Unknown	15 (< 0.1)	0 (0)
*BMI*		
< 25.0	18,331 (38)	83 (35)
25.0–29.9	15,205 (32)	74 (31)
≥ 30.0	14,361 (30)	82 (34)
Unknown	16 (< 0.1)	0 (0)
*Personal attained education level*		
High school, GED, or less	7290 (15)	29 (12)
Some college or associate or technical degree	16,161 (34)	80 (33)
Bachelor’s degree or more	24,450 (51)	130 (54)
Unknown	12 (< 0.1)	0 (0)
*Annual household income*		
< $50,000	12,228 (26)	61 (26)
$50,000-$99,999	19,552 (41)	94 (39)
≥ $100,000	16,133 (34)	84 (35)

*BMI: body mass index (kg/m^2^)

*GED: General Educational Development

### Associations with Childhood and Adolescent Factors

Table 2 shows multivariable-adjusted HRs for childhood and adolescent factors and DTC incidence. Being taller than peers at age 10 was associated with DTC incidence (HR = 1.41; 95%CI 1.06–1.89). Women who were either lighter (HR = 1.37, 95%CI 0.97–1.91) or heavier (HR = 1.28, 95%CI 0.96–1.71) than peers during teen years had higher DTC incidence, although the risk estimates did not reach statistical significance. Ever not having enough to eat during childhood was associated with a higher DTC incidence (HR = 1.67, 95%CI 1.15–2.43). Greater household education at age 13 was associated with a non-significantly lower incidence of DTC (highest education level at or above a Bachelor’s degree versus high school, GED, or less: HR = 0.75, 95% CI 0.55–1.03). Other anthropometric, reproductive, lifestyle, and socioeconomic factors were not associated with DTC incidence.

**Table 2:** Association between childhood and adolescent factors and DTC incidence in the Sister Study cohort

Characteristic		Multivariable models			
	Total cohort, *n* (%)	DTC cases, *N* (%)	Person-years	HR	95% CI
**Growth and reproductive factors**					
*Relative weight to peers at age 10*					
Lighter	16,793 (35)	80 (33)	203,318	1.03	0.77, 1.38
Same weight	22,324 (47)	105 (44)	271,626	1	—
Heavier	8644 (18)	52 (22)	106,503	1.25	0.90, 1.75
*Height relative to peers at age 10*					
Shorter	12,285 (26)	53 (22)	150,199	0.94	0.68, 1.32
Same height	21,984 (46)	99 (41)	266,794	1	—
Taller	13,556 (28)	87 (36)	165,201	1.41	1.06, 1.89
*Weight relative to peers during teen years*					
Lighter	16,658 (35)	90 (38)	201,757	1.28	0.96, 1.71
Same weight	22,461 (47)	97 (41)	273,839	1	—
Heavier	8,735 (18)	52 (22)	106,906	1.37	0.97, 1.91
*Age at breast development*					
< 11 years of age	6352 (13)	32 (13)	76,904	1.04	0.71, 1.52
11–13 years of age	32,749 (68)	160 (67)	399,844	1	—
≥ 14 years of age	8233 (17)	44 (18)	99,796	1.11	0.79, 1.55
Age at breast development (continuous, imputed, median, IQR)	12.0 (11.0, 13.0)	239 (100)	583,135	1.00	0.94, 1.06
*Age at menarche*					
< 12 years of age	9,735 (20)	55 (23)	117,128	1.16	0.85, 1.59
12–13 years of age	26,875 (56)	134 (56)	328,470	1	—
≥ 14 years of age	11,246 (24)	50 (21)	136,821	0.89	0.64, 1.23
Age at menarche (continuous, imputed, median, IQR)	13.0 (12.0, 13.0)	239 (100)	583,135	0.98	0.91, 1.06
*Age started using hormonal birth control*					
Started after age 20	26,046 (54)	130 (54)	315,004	1	—
Started before age 20	20,864 (44)	105 (44)	256,147	0.94	0.72, 1.24
**Lifestyle factors**					
*Physical activity between age 5 and 20 (MET-hours/week)*					
< 21 MET-hours/week	41,526 (87)	209 (87)	506,471	1	—
21- < 42 MET-hours/week	3964 (8)	22 (9)	47,968	1.09	0.70, 1.69
≥ 42 MET-hours/week	2100 (4)	5 (2)	25,155	0.47	0.19, 1.14
Physical activity between age 5 and 20 (MET-hours/week, continuous, imputed, median, IQR)	1.8 (0.0, 10.0)	239 (100)	583,135	0.99	0.98, 1.00
*Age started drinking regularly*					
Stated after age 20	14,977 (31)	66 (28)	178,111	1	—
Started before age 20	32,905 (69)	173 (72)	404,665	1.09	0.81, 1.47
Number of drinks per year between age 5 and 20 (drinks/year)					
0 drink/year	15,136 (32)	66 (28)	179,860	1	—
< 60 drinks/year	14,083 (29)	74 (31)	174,500	1.11	0.79, 1.55
60–229 drinks/year	8983 (19)	38 (16)	111,049	0.86	0.57, 1.30
≥ 230 drinks/year	5269 (11)	33 (14)	64,920	1.25	0.80, 1.93
*Age started smoking and number of pack-years*					
Started after age 20	32,020 (67)	156 (65)	393,059	1	—
Started before age 20	15,874 (33)	83 (35)	189,859	1.11	0.85, 1.45
Started before age 20, ≤ 5 pack-years	14,852 (31)	78 (33)	177,875	1.11	0.85, 1.46
Started before age 20, > 5 pack-years	914 (2)	5 (2)	10,802	1.15	0.47, 2.80
*Total years of secondhand smoking under age 18 from caregiver or household member*					
No secondhand smoking during childhood	13,867 (29)	72 (30)	169,783	1	—
≤ 10 years	6258 (13)	30 (13)	76,550	0.94	0.61, 1.44
> 10 years	26,919 (56)	133 (56)	326,740	0.99	0.74, 1.32
**Socioeconomic factors**					
*Family income while growing up*					
Well off	3066 (6)	13 (5)	37,935	0.78	0.44, 1.37
Middle income	28,582 (60)	152 (64)	351,548	1	—
Low income	12,350 (26)	56 (23)	148,793	0.91	0.67, 1.24
Poor	3799 (8)	17 (7)	43,676	0.96	0.58, 1.61
*Ever not having enough to eat during childhood*					
No	43,388 (91)	206 (86)	531,069	1	—
Yes	4492 (9)	33 (14)	51,706	1.67	1.15, 2.43
*Highest household education level at age 13*					
Highschool or GED or less	25,729 (54)	134 (56)	307,924	1	—
Some college or associate or technical degree	8889 (19)	45 (19)	109,360	0.93	0.66, 1.30
Bachelor’s degree or higher	12,736 (27)	56 (23)	159,924	0.75	0.55, 1.03
*Family type at age 13*					
Two parents	42,700 (89)	220 (92)	522,033	1	—
Single parent	4669 (10)	15 (6)	54,984	0.68	0.40, 1.15
*Childhood urbanicity*					
Urban or suburban areas	24,001 (50)	126 (53)	294,128	1	—
Small town or rural areas	23,207 (48)	111 (46)	280,828	0.93	0.72, 1.20

*HR Hazard Ratio, *CI* Confidence Interval, *DTC* Differentiated thyroid cancer, *GED* General Educational Development, *IQR* Interquartile range, *MET* Metabolic Equivalent of Task, Multivariable models used attained age as the timescale and were adjusted for self-identified race/ethnicity. Results for “Unknown” categories are not shown. The number of observations within the “Unknown” categories for each variable is as follows: Weight relative to peers at age 10 (n = 152), Height relative to peers at age 10 (n = 88), Weight relative to peers during teen years (n = 59), Age at breast development (n = 579), Age at menarche (n = 57), Age started using hormonal birth control (n = 1003), Physical activity between age 5 and 20 (METhours/week) (n = 323), Age started drinking regularly (n = 31), Number of drinks per year between age 5 and 20 (drinks/year) (n = 4442), Total years of secondhand smoking under age 18 from caregiver or household member (n = 869), Family income while growing up (n = 116), Ever not having enough to eat during childhood (n = 33), Highest household education level at age 13 (n = 559), Household composition at age 13 (n = 544), Childhood urbanicity (n = 705)

Because early-life socioeconomic characteristics may be interrelated and could influence childhood and adolescent body size, we performed the following sensitivity analyses: (1) a model adjusted simultaneously for height relative to peers at age 10, weight relative to peers during teen years, ever not having enough to eat during childhood, and highest household education level at age 13, and (2) another model adjusted for family income while growing up, household composition at age 13, and childhood urbanicity in addition to all previously mentioned variables. The risk estimates were largely unchanged in these mutually adjusted models, except for a more pronounced association for ever not having enough to eat during childhood (HR = 1.90, 95%CI 1.26–2.90) (Table 3).

**Table 3:** Mutually adjusted associations between for selected factors and DTC incidence

Characteristic	DTC cases	Model 1		Model 2	
		HR	95% CI	HR	95% CI
*Height relative to peers at age 10*					
Shorter	53	0.9	0.64, 1.26	0.89	0.64, 1.25
Same height	99	1	—	1	—
Taller	87	1.39	1.04, 1.86	1.38	1.03, 1.85
Unknown	0	—	—	—	—
*Weight relative to peers during teen years*					
Lighter	90	1.29	0.96, 1.72	1.29	0.97, 1.73
Same weight	97	1	—	1	—
Heavier	52	1.3	0.93, 1.83	1.31	0.93, 1.83
Unknown	0	—	—	—	—
*Ever not having enough to eat during childhood*					
No	206	1	—	1	—
Yes	33	1.58	1.08, 2.31	1.90	1.25, 2.88
Unknown	0	—	—	—	—
*Highest household education level at age 13*					
Highschool or GED or less	134	1	—	1	—
Some college or associate or technical degree	45	0.94	0.67, 1.32	0.89	0.63, 1.26
Bachelor’s degree or higher	56	0.77	0.56, 1.07	0.71	0.51, 1.00
Unknown	4	1.8	0.66, 4.88	1.72	0.62, 4.77

*HR Hazard Ratio, *CI* Confidence Interval, *DTC* Differentiated thyroid cancer, *GED* General Educational Development, *IQR* Interquartile range, *MET* Metabolic Equivalent of Task, Model 1 was simultaneously adjusted for attained age (timescale), self-identified race/ethnicity, height relative to peers at age 10, and weight relative to peers during teen years, ever not having enough to eat during childhood, and highest household education level at age 13. Model 2 was simultaneously adjusted for all covariates of model 1 and family income while growing up, family type at age 13, and childhood urbanicity

### Effect Modification

**Fig. 1:**
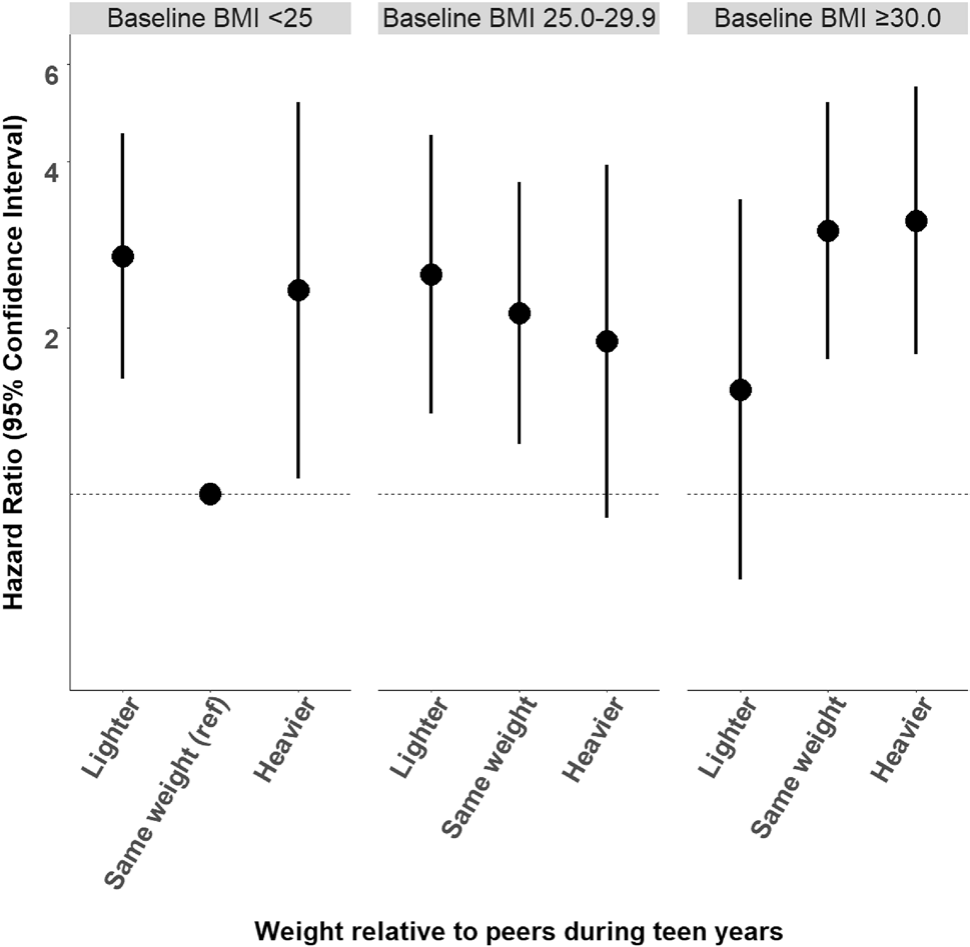
The joint associations of weight relative to peers during teen years and baseline BMI with differentiated thyroid cancer incidence

Most associations were similar across baseline socioeconomic status and BMI strata (p-interactions > 0.05, Supplementary Fig. 1–4). The only exception was weight relative to peers during teen years, where we observed a significant interaction by baseline BMI (p-interaction = 0.03). Specifically, positive associations for weight relative to peers during teen years (lighter or heavier vs. same weight) were most pronounced in women with a baseline BMI under 25 (Fig. 1).

### Secondary Analyses

Associations with weight relative to peers during teen years, ever not having enough to eat during childhood, and highest household education level at age 13 were similar for early- and late-onset DTC. The association for height relative to peers at age 10 appeared to be stronger for early- versus later-onset DTC (Supplementary Table 1). Restricting to papillary thyroid cancer (Supplementary Table 1), medically confirmed cases, or cases with complete data (Supplementary Table 2) did not materially change results.

The E-values for the associations between DTC incidence and being taller than peers at age 10, ever not having enough to eat during childhood, and having a bachelor’s degree or higher as the highest household education level at age 13 were 2.18, 2.73, and 1.99, respectively, indicating that an HR of at least 2 to threefold for any unmeasured confounders associated with early-life factors and DTC would be necessary to explain the observed associations (Supplementary Table 2).

## Discussion

Our study contributes to the limited evidence on early-life factors and DTC incidence. We found higher DTC incidence to be associated with several measures of early-life body size, specifically, taller height than peers at age 10, and lighter or heavier weight than peers during teen years (particularly for women with a BMI less than 25 at study baseline). Not having enough to eat during childhood was associated with increased incidence and greater household education was associated with lower DTC incidence. We found no clear associations for early-life reproductive or hormonal factors, such as age at breast development or menarche or use of oral contraceptives before age 20.

## Growth and Socioeconomic Factors

Few other longitudinal studies have examined early-life body size in relation to DTC incidence. In a population-based cohort of children and adolescents in Denmark with a median follow-up of 39 years, taller height and greater BMI measured at every age between 7 and 13 were associated with higher adult thyroid cancer incidence. The associations for BMI were generally stronger for those diagnosed with DTC at younger ages [18]. Similarly, data from an Israeli nationwide cohort showed positive associations between greater height [19] and BMI [20] measured at ages 16–18 and thyroid cancer incidence after mean follow-up periods of 10 and 19 years, respectively. However, these studies did not account for adult anthropometric factors, which limits the interpretation of body size effects at different life stages [18–20]. Our study is the first, to our knowledge, to examine the joint associations of childhood height and weight and adulthood body size on thyroid cancer incidence. We observed consistently positive associations for being taller compared to peers during childhood and adolescence across adult BMI categories, while being heavier than peers was associated with higher DTC incidence only among women with an adult BMI < 25. These findings suggest that early-life body size may have a long-term influence on DTC incidence independent of adulthood body size.

Our observations of a higher DTC incidence among women who reported being lighter than peers during childhood and adolescence may point to a role of early-life nutrition or socioeconomic conditions. Before the U.S. obesity epidemic began in the late 1970s, [37] being lighter than peers and not having enough to eat in early life could have indicated lower socioeconomic status [38], which in turn could shape exposures to other environmental and lifestyle risk factors for cancer [27]. However, in our study, these associations either persisted or were more pronounced after adjusting for both childhood and adult socioeconomic factors, suggesting that socioeconomic status may not be the primary driver. Suboptimal nutritional exposures in early life, including nutritional deficiencies and physiological adaptive responses, may offer another explanation, although the role of specific dietary factors in thyroid cancer development is not well-understood [39]. Suboptimal nutrition in early life may trigger adaptive epigenetic changes [40], hormonal imbalances, and growth disruptions [41], specifically variations in growth hormone and IGF-1 levels. In addition to higher IGF-1 levels, early-life caloric restriction also has been associated with taller stature and heavier weight [42, 43]. IGF-I has been suggested to influence carcinogenesis given its role in cell proliferation, differentiation, metabolism, apoptosis, and angiogenesis [44]. Experimental studies have found that IGF-1 is more highly expressed in thyroid cancer than in normal tissues and benign lesions [45]. Moreover, there is evidence of increased risk of thyroid cancer in individuals with elevated growth hormone/IGF-1 signaling, such as patients with acromegaly [46, 47]. Recent large European population-based studies have showed a positive association between adult IGF-1 levels and subsequent thyroid cancer incidence [48, 49]. However, the underlying mechanisms linking IGF-1 and growth hormone levels in early life with cancer later in life are not well-understood. Therefore, studies with objective longitudinal measurements of early-life anthropometric factors, IGF-1, and growth hormone levels are warranted.

## Reproductive Factors

In the current study, we did not find clear associations for factors reflecting early or delayed exposure to endogenous sex steroid hormones early in life, including age at menarche and age at breast development, in agreement with some [24, 25], but not all [22, 23], previous studies. We also did not observe associations for use of hormonal birth control in adolescence. Previous studies have shown inconsistent results on the associations for hormonal birth control [22–25], which could be due to the variability in the duration of use and the evolving formulations of birth controls over the years. In the Sister Study, most individuals (99.4%) who started hormonal birth control before age 20 used combined oral contraceptive. These individuals were likely exposed to early formulations in the 1960s with high dose of estrogen up to 150 μg of mestranol [50], compared to current formulations, which contain as low as 20 μg of ethinyl estradiol [51]. Experimental studies have demonstrated that estrogen not only directly stimulates growth in both benign and malignant thyroid cells, but potentially simulates the tyrosine kinase signaling pathways MAPK and PI3K and plays a role in angiogenesis regulation for thyroid cancer. [12]

## Strengths and Limitations

The major strengths of this study include the large sample size, long follow-up, and wide range of childhood and adolescent exposures. Due to the prospective design, early-life factors were reported prior to cancer diagnosis, thus eliminating differential recall between cases and non-cases. Non-differential exposure misclassification tends to drive associations toward the null rather than induce spurious positive findings. While personal recall of childhood and adolescent exposures at baseline may have introduced exposure misclassification, validation studies show that adults can report their relative and recalled body size with moderate to strong accuracy even decades later, with correlations to measured indices ranging from 0.56 to 0.84 [52]. To further ensure temporality between the exposures and outcome of interest in this analysis, women with a baseline history of DTC were excluded. While this exclusion could potentially lead to missing associations for early-onset DTC, most associations were similar for early- versus late-onset DTCs, and the median age at baseline (55.4 years) corresponded to the peak of the age-at-diagnosis curve for DTC incidence [5]. Although we were missing information on potential risk factors for thyroid cancer, including genetic factors, childhood exposure to ionizing radiation, other unknown or suspected environmental risk factors, dietary intake, or access to thyroid cancer screening [2], such factors are unlikely to be major confounders, as they are not expected to be strongly associated with the early-life factors investigated in the current study. Also, our assessment of E-values showed that their effects would have to be at least 2- to threefold to explain our results. With only 239 total incident DTC cases, statistical power was limited in certain analyses, particularly stratified or subgroup analyses.

Finally, the results may have limited generalizability. As childhood and adolescence for most study subjects occurred between the 1930s and 1970s, some of our results may not be generalizable to modern-day populations. For example, obesogenic diet, lifestyle, and environmental factors have become more commonplace since the late 1970s, particularly in the United States [53, 54]. Also, as the Sister Study enrolled women having a sister diagnosed with breast cancer, the results may not be generalizable to men or women without a family history of breast cancer. However, there is limited evidence to suggest that family history of breast cancer influences thyroid cancer incidence.

## Conclusion

In conclusion, the current study supports the influence of early-life exposures, including relative body size, ever not having enough to eat, and higher household education levels, on subsequent DTC incidence. These findings offer further clues into the etiology of DTC, including a possible role of early-life growth and nutrition.

## Supplementary Information

Below is the link to the electronic supplementary material.

Supplementary file1 (DOCX 188 KB)

## Data Availability

Data used in this article are available as described on the Sister Study website (The Sister Study: Collaborations and Data Requests, nih.gov) or by request via the Sister Study tracking and review system (www.sisterstudystars.org; registration required). Computing code can be requested from the corresponding author.
