# Supplementary material for: Childhood and Adolescent Factors and Thyroid Cancer Incidence in Adult Women in the Sister Study Cohort
Source: Cancer Causes Control. 2026 Feb 20;37(3):53. doi: 10.1007/s10552-025-02085-1 (PMC12923491; doi:10.1007/s10552-025-02085-1)
Supplement: Supplementary file 1 — Supplementary file1 (DOCX 188 KB) [file 10552_2025_2085_MOESM1_ESM.docx]

**Supplementary materials**

## Supplementary Figure 1: Association between height relative to peers at age 10and differentiated thyroid cancer incidence in the Sister Study participants stratified according to potential modifying factors at baseline


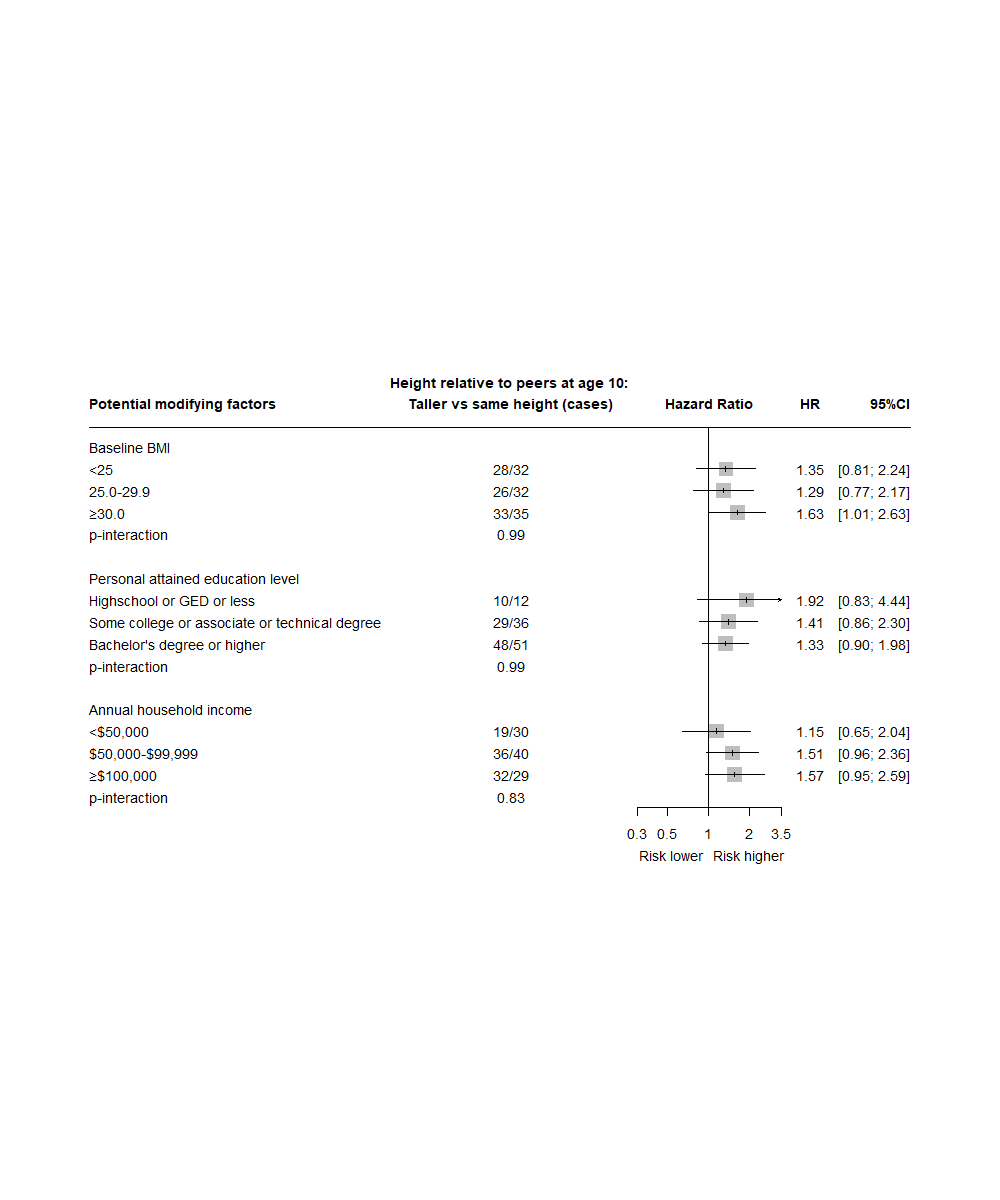


## Supplementary Figure 2: Association between weight relative to peers during teen years and differentiated thyroid cancer incidence in the Sister Study participants stratified according to potential modifying factors at baseline


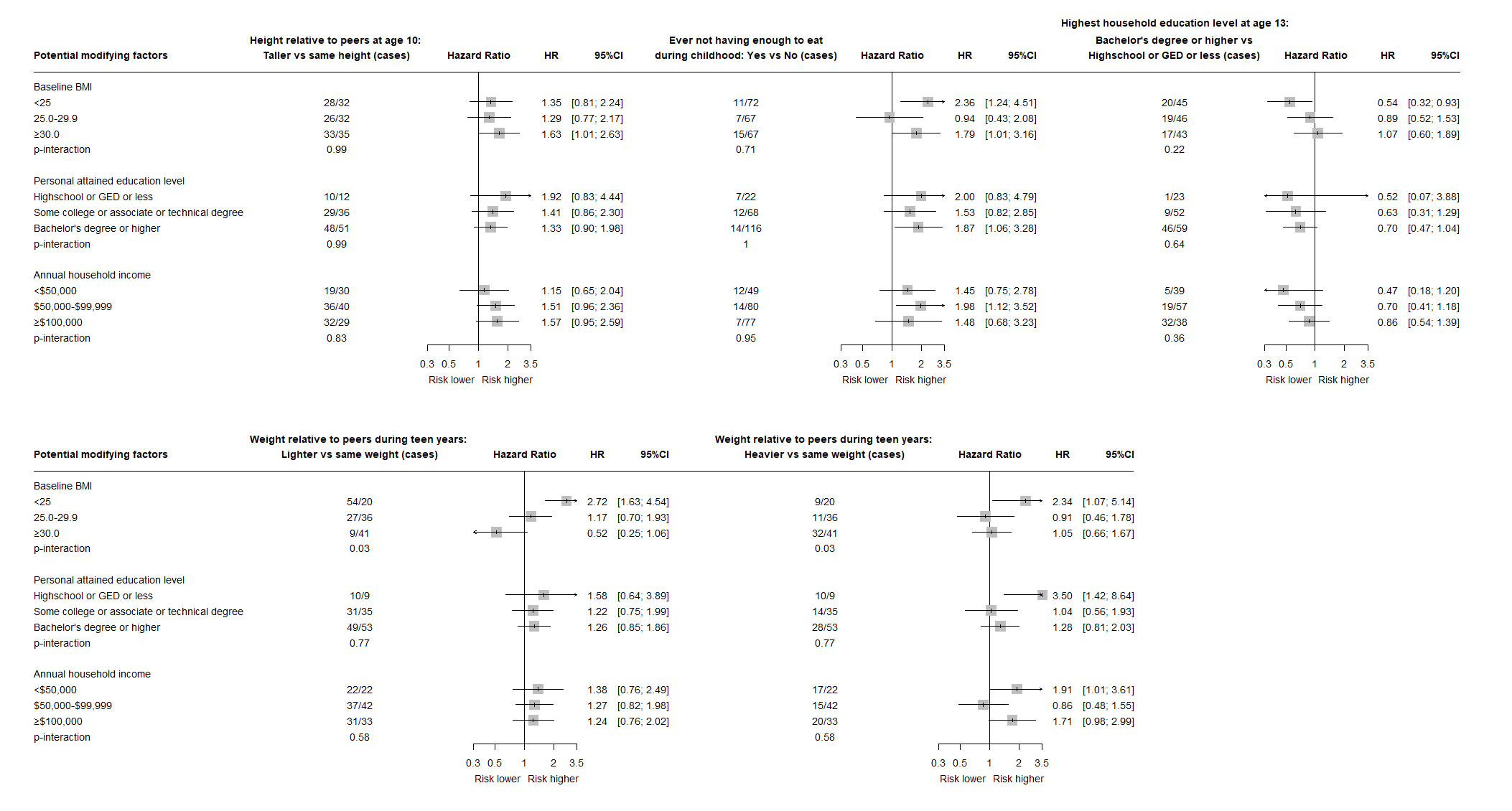


## Supplementary Figure 3: Association between ever not having enough to eat during childhood and differentiated thyroid cancer incidence in the Sister Study participants stratified according to potential modifying factors at baseline


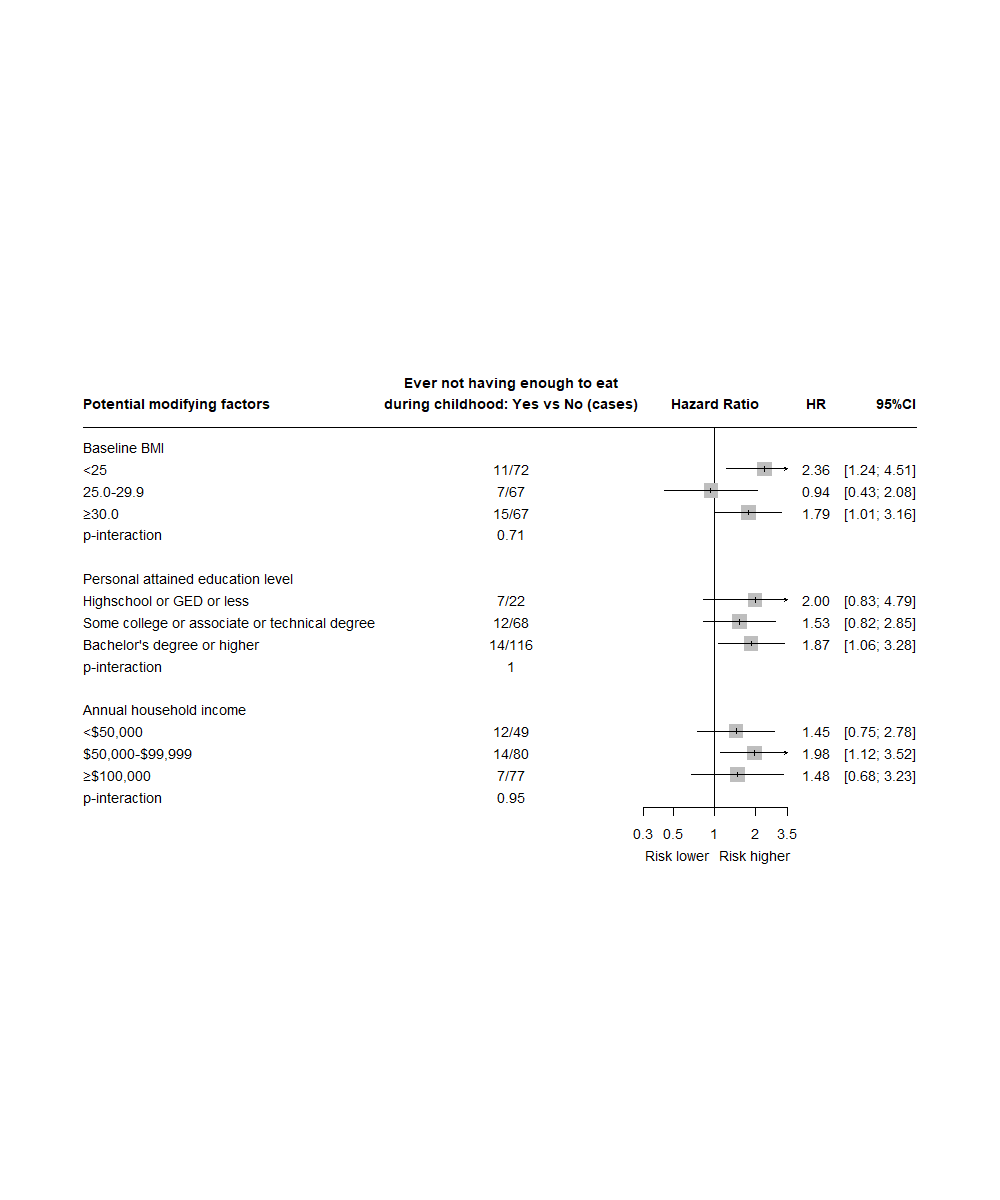


## Supplementary Figure 4: Association between highest household education level at age 13 and differentiated thyroid cancer incidence in the Sister Study participants stratified according to potential modifying factors at baseline


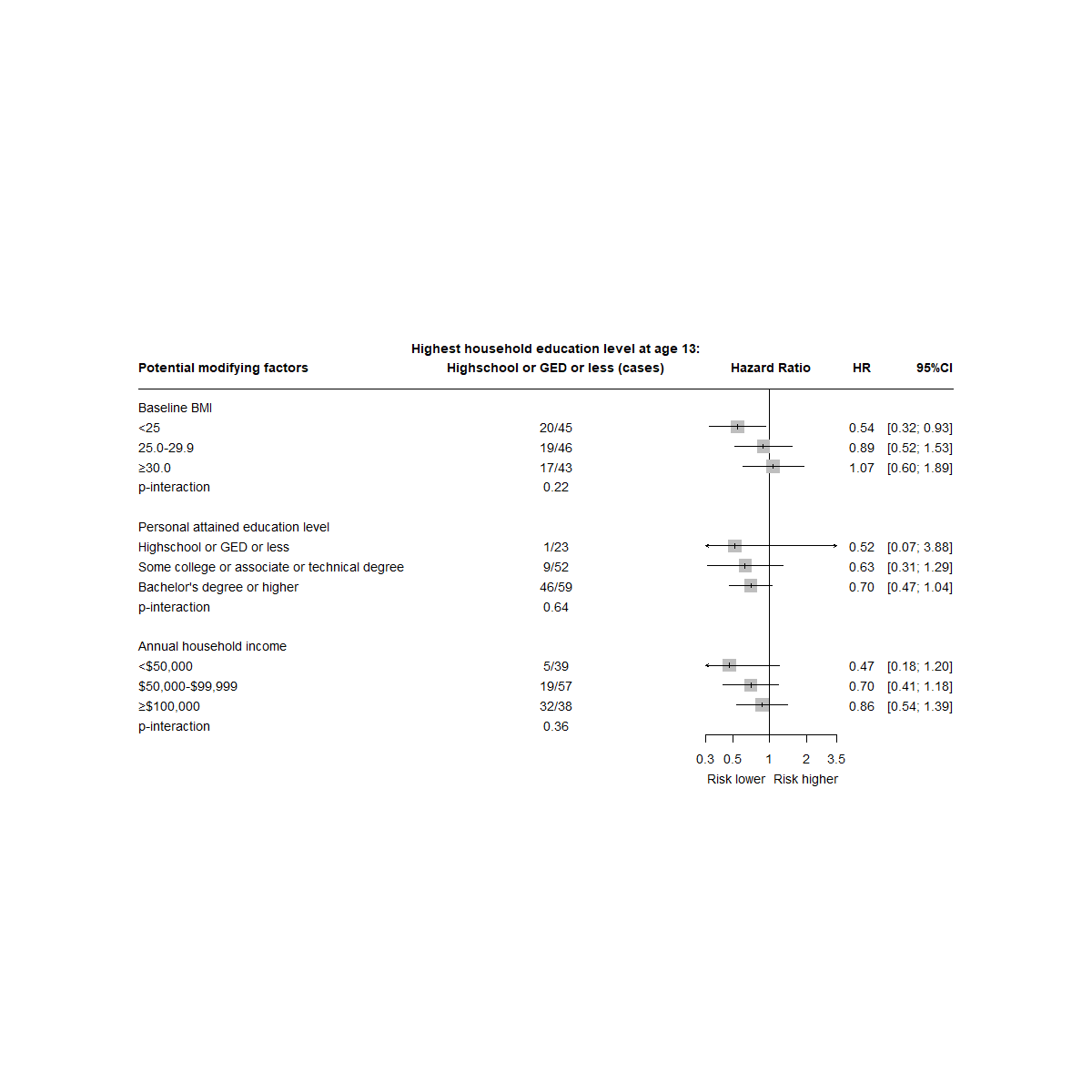


## Supplementary table 1: Association between childhood and adolescent factors and differentiated thyroid cancer incidence in the Sister Study participants stratified by cancer subtypes

| **Characteristic** | **Early-onset cancers** | | | **Late-onset cancers** | | | | **Papillary thyroid cancer** | | |
| --- | --- | --- | --- | --- | --- | --- | --- | --- | --- | --- |
|  | **DTC cases** | **HR** | **DTC cases** | | **DTC cases** | **DTC cases** | **95% CI** | **DTC cases** | **HR** | **95% CI** |
| Height relative to peers at age 10 | | | |  | |  |  |  |  |  |
| Shorter | 9 | 1.18 | 41 | | 41 | 41 | 0.63, 1.31 | 43 | 1.05 | 0.72, 1.54 |
| Same height | 13 | 1 | 66 | | 66 | 66 | — | 72 | 1 | — |
| Taller | 17 | 2.05 | 57 | | 57 | 57 | 0.96, 1.81 | 60 | 1.34 | 0.95, 1.88 |
| Unknown^1^ | 0 | — | 0 | | 0 | 0 | — | 0 | — | — |
| Weight relative to peers during teen years | | |  | |  |  |  |  |  |  |
| Lighter | 14 | 1.17 | 67 | | 67 | 67 | 0.95, 1.78 | 71 | 1.52 | 1.08, 2.12 |
| Same weight | 16 | 1 | 60 | | 60 | 60 | — | 65 | 1 | — |
| Heavier | 9 | 1.26 | 37 | | 37 | 37 | 0.96, 2.00 | 39 | 1.53 | 1.03, 2.27 |
| Unknown^1^ | 0 | — | 0 | | 0 | 0 | — | 0 | — | — |
| Ever not having enough to eat during childhood | | | |  | |  |  |  |  |  |
| No | 32 | 1 | 146 | | 146 | 146 | — | 155 | 1 | — |
| Yes | 7 | 2.05 | 18 | | 18 | 18 | 1.05, 2.42 | 20 | 1.36 | 0.85, 2.19 |
| Unknown^1^ | 0 | — | 0 | | 0 | 0 | — | 0 | — | — |
| Highest household education level at age 13 | | | |  | |  |  |  |  |  |
| Highschool or GED or less | 21 | 1 | 92 | | 92 | 92 | — | 98 | 1 | — |
| Some college or associate or technical degree | 9 | 1.08 | 29 | | 29 | 29 | 0.61, 1.30 | 31 | 0.86 | 0.58, 1.30 |
| Bachelor's degree or higher | 8 | 0.58 | 40 | | 40 | 40 | 0.57, 1.13 | 43 | 0.78 | 0.54, 1.13 |
| Unknown^1^ | 0 | — | 3 | | 3 | 3 | — | 3 | — | — |
| HR = Hazard Ratio, CI = Confidence Interval. The risk estimates are shown when there were at least 5 cases.  DTC = Differentiated thyroid cancer  Multivariable models used attained age as the timescale and were adjusted for self-identified race/ethnicity  *^1^*Results for “Unknown” categories of are not shown | | | | | | | | | | |

## Supplementary table 2: Association between childhood and adolescent factors and differentiated thyroid cancer incidence in the Sister Study participants: Sensitivity analyses

| **Characteristic** | **Medically confirmed DTC cases^1^** | | | **Complete case analysis** | | | **E-values** | | |  |  |
| --- | --- | --- | --- | --- | --- | --- | --- | --- | --- | --- | --- |
|  | **DTC cases** | **HR** | **95% CI** | **DTC cases** | **HR** | **95% CI** | | **E-values for HR** | **E-values for CI^2^** | |  |
| Height relative to peers at age 10 |  |  |  |  |  |  | |  |  | |  |
| Shorter | 43 | 1.05 | 0.72, 1.54 | 53 | 0.94 | 0.68, 1.32 | | 1.31 | 1 | |  |
| Same height | 72 | 1 | — | 99 | 1 | — | |  |  | |  |
| Taller | 60 | 1.34 | 0.95, 1.88 | 87 | 1.41 | 1.06, 1.89 | | 2.18 | 1.31 | |  |
| Unknown | 0 | — | — | 0 |  |  | |  |  | |  |
| Weight relative to peers during teen years |  |  |  |  |  |  | |  |  | |  |
| Lighter | 71 | 1.52 | 1.08, 2.12 | 90 | 1.28 | 0.96, 1.71 | | 1.88 | 1 | |  |
| Same weight | 65 | 1 | — | 97 | 1 | — | |  |  | |  |
| Heavier | 39 | 1.53 | 1.03, 2.27 | 52 | 1.37 | 0.97, 1.91 | | 2.07 | 1 | |  |
| Unknown | 0 | — | — | 0 |  |  | |  |  | |  |
| Ever not having enough to eat during childhood |  |  |  |  |  |  | |  |  | |  |
| No | 155 | 1 | — | 206 | 1 | — | |  |  | |  |
| Yes | 20 | 1.36 | 0.85, 2.19 | 33 | 1.67 | 1.15, 2.43 | | 2.73 | 1.56 | |  |
| Unknown | 0 | — | — | 0 |  |  | |  |  | |  |
| Highest household education level at age 13 |  |  |  |  |  |  | |  |  | |  |
| Highschool or GED or less | 98 | 1 | — | 134 | 1 | — | |  |  | |  |
| Some college or associate or technical degree | 31 | 0.86 | 0.58, 1.30 | 45 | 0.93 | 0.66, 1.31 | | 1.38 | 1 | |  |
| Bachelor's degree or higher | 43 | 0.78 | 0.54, 1.13 | 56 | 0.76 | 0.55, 1.04 | | 1.99 | 1.22 | |  |
| Unknown | 3 | — | — | 0 |  |  | | — | — | |  |
| HR = Hazard Ratio, CI = Confidence Interval. The risk estimates are shown when there were at least 5 cases. | | | | | | | | | | | |
| DTC = Differentiated thyroid cancer | | | | | | | | | | | |
| Multivariable models used attained age as the timescale and were adjusted for self-identified race/ethnicity | | | | | | | | | | | |
| *^1^* Results for “Unknown” categories of are not shown | | | | | | | | | | | |
| ^2^ E-values for the limit of the confidence interval closest to the null | | | | | | | | | | | |
